# The preoperative SUVmax for ^18^F-FDG uptake predicts survival in patients with colorectal cancer

**DOI:** 10.1186/s12885-015-1991-5

**Published:** 2015-12-21

**Authors:** Debing Shi, Guoxiang Cai, Junjie Peng, Dawei Li, Xinxiang Li, Ye Xu, Sanjun Cai

**Affiliations:** Department of Colorectal Surgery, Fudan University Shanghai Cancer Center, Shanghai, 200032 China; Department of Oncology, Shanghai Medical College, Fudan University, Shanghai, 200032 China

**Keywords:** Colorectal cancer, ^18^F-FDG, PET/CT, SUVmax, Histopathologic, Immunohistochemical

## Abstract

**Background:**

The study was to investigate whether ^18^F-fluorodeoxyglucose (^18^F-FDG) uptake, analyzed by positron emission tomography (PET), can be used preoperatively to predict survival in Chinese patients with colorectal carcinoma.

**Methods:**

A prospectively maintained colorectal cancer database was retrospectively reviewed between June 2009 and December 2011. All included patients had been newly diagnosed with colorectal cancer (of various stages) and evaluated by ^18^F-FDG-PET/computed tomography (CT) within the 2 weeks preceding surgery. Univariate and multivariate analyses were used to determine whether the maximal standardized uptake value (SUVmax) and various clinicopathological and immunohistochemical factors were correlated with survival. Receiver operating characteristics (ROC) curve and Kaplan-Meier survival curve analyses were used to explore whether SUVmax could predict survival in these patients.

**Results:**

A total of 107 patients were enrolled in the study (mean age, 59.26 ± 12.66 years; 66.35 % males), with 77 surviving to the end of follow-up (average 60 months). Univariate analysis indicated that tumor size, TNM stage, nodal metastasis, the ratio of metastasized nodes to retrieved nodes, cyclin D1 immunostaining and SUVmax correlated with survival (*P* < 0.05). Multivariate analysis showed that only TNM stage and SUVmax were associated with survival (*P* < 0.05). ROC curve analysis determined the optimal SUVmax cutoff for predicting survival to be 11.85 (sensitivity, 73.3 %; specificity, 75.3 %). Survival was significantly longer in patients with preoperative SUVmax ≤11.85 (*P* < 0.001, log-rank test).

**Conclusions:**

SUVmax, measured by ^18^F-FDG-PET/CT, provides a useful preoperative prognostic factor for patients with colorectal cancer.

## Background

Colorectal cancer is a common malignancy in the Western world, and its incidence continues to increase in China [1, 2]. Generally, patients are diagnosed with colorectal cancer in the sixth and seventh decades of life, with most lesions occurring in the sigmoid (30 %), rectum (25 %) and cecum (25 %) [1]. More than 50 % of patients with colorectal cancer will have developed metastases by the time of diagnosis [3–6], most commonly to the liver and lungs, highlighting the need for new markers that will more accurately predict prognosis.

Imaging modalities are frequently used in the screening, staging and surveillance of colorectal cancer. ^18^F-fluorodeoxyglucose (^18^F-FDG) positron emission tomography (PET) has proven particularly useful in the clinical staging and restaging of metastases or local recurrence of colorectal cancer [7]. However, the diagnostic accuracy of FDG/PET is limited by nonspecific colonic uptake of FDG that is unrelated to malignancy, as a result of physiologic processes, inflammation or colonic adenomas [8]. Accumulation of FDG in a tumor is based on enhanced glycometabolism, and there is substantial evidence that FDG uptake in tumor cells correlates with tumor growth rate, the potential for aggressive behavior, and prognosis [9]. Thus, FDG-PET may be used before surgery to assess tumor metabolism. The standardized uptake value (SUV) is the semiquantitative parameter most commonly used in current clinical practice to assess the degree of FDG accumulation.

Many clinicopathological factors have been reported to be potential prognostic markers for colorectal cancer, including lymph node status, presence of metastases and differentiation status [10]. In addition, the expressions of various endogenous proteins, detectable using immunohistochemical (IHC) techniques, have been suggested by some (but not all) studies to correlate with the proliferative capacity, invasive potential and/or prognosis of colorectal cancer. These proteins include Ki-67, proliferating cell nuclear antigen (PCNA), cyclin D1 (CCND1), and nm23 (a nucleoside diphosphate kinase) [11–13].

Unfortunately, these pathological and IHC indexes can only be assessed after surgery. Preoperative prediction of patient prognosis would have numerous benefits, allowing better selection of patients for neoadjuvant radiochemotherapy to downstage their disease, and increasing the feasibility of sphincter-sparing surgery. In addition, preoperative diagnostic markers could help to evaluate the chemosensitivity of the cancer. The present study has investigated whether ^18^F-FDG uptake, analyzed by PET, can be used preoperatively to predict survival in Chinese patients with colorectal carcinoma. Our findings could help to extend the use of FDG-PET/CT as a technique for preoperative prediction of prognosis in patients with colorectal cancer.

## Methods

### Patients

A prospectively maintained colorectal cancer database was retrospectively reviewed between June 2009 and December 2011. The patients selected for this study had been newly diagnosed with colorectal cancer (of various stages) and evaluated further by ^18^F-FDG-PET/CT within the 2 weeks preceding surgery. Patients were excluded if they were hyperglycemic (>9 mmol/L) on the day of the PET/CT investigation, or had received any therapeutic or major surgical interventions before examination. All patients taking metformin stopped this drug for a week before examination. Follow-up data were collected, and each patient allocated into one of two groups (survivor or deceased) according to their clinical outcome at an average follow-up time of 60 months. This study was approved by the Ethics Committee of the Shanghai Cancer Center, Fudan University, P.R. China. Written informed consent was obtained from each patient for original data entry. The Committee waived the need for individual consent for subsequent studies carried out using this database.

### Data collection

*Clinical data:* The following characteristics were extracted from the clinical records: patient gender, age, and tumor size (cm). Tumor size was estimated by measuring the maximal diameter of the invasive component of the tumor.

*Maximum standardized uptake value:* All patients underwent ^18^F-FDG PET/CT scans (Biograph, 16HR; Siemens, Germany) during the 2 weeks immediately preceding surgery. Patients fasted for at least 4 h before the ^18^F-FDG PET/CT study, and blood glucose analysis of capillary blood samples was undertaken 1 h before injection of FDG. Patients received 0.2 mCi/kg (74 MBq/kg) of ^18^F-FDG intravenously, via a vein in the arm, and then were allowed to rest until the start of the scan. With the patient in the supine position, three-dimensional (3D) PET acquisition was performed from the skull to the upper thighs, with 5-7 bed positions per 2 min. The images were reconstructed with a standard algorithm provided by the manufacturer. The CT component (120 kV; 300 mA, with the electric current controlled automatically by the CareDose4D software according to cine-oriented image; 5-mm slice thickness, inter-slice spacing and reconstruction) was performed without intravenous contrast or bowel preparation, for the purpose of correction of attenuation and lesion localization. PET data were acquired in the same anatomic location. The region of interest (ROI) was delineated according to the margin of the mass on the PET image. The FDG activity was measured by calculating the maximal SUV (SUVmax) in the attenuation-corrected PET data. The SUV was calculated using the following formula: SUV = activity in the region of interest (MBq/mL)/injected dose (MBq)/body weight (kg).

*Pathological data:* Tissue samples were processed using a standard protocol [14]. Histological grade and type, invasion depth and lymphovascular or nerve invasion were determined for each patient by at least two observers, who were unaware of the results of the PET/CT studies. The pathological results served as the reference standard, and the tumor stage was classified according to the seventh edition of the TNM staging system for colorectal cancer. In the present study, the following pathological factors were measured: TNM stage, histological type, differentiation degree, nodal metastasis status, and the ratio of nodal metastasis to total lymph nodes retrieved. TNM stage was classified into 6 subgroups: Tis, I, IIA, IIB, III and IV; histologic type was divided into 6 subgroups: adenocarcinoma, mucinous adenocarcinoma; signet ring cell carcinoma, adenocarcinoma with a component of mucinous adenocarcinoma, adenocarcinoma with a component of signet ring cell carcinoma, and others; and differentiation degree was classified into 6 subgroups: well differentiated, well or moderately differentiated, moderately differentiated, moderately or poorly differentiated, poorly differentiated, and undifferentiated.

*Immunohistochemical data:* Consecutive sections with a thickness of 4 μm were cut from representative paraffin-embedded tumor blocks. IHC staining was performed on an automated platform (BenchMark XT, Ventana Medical Systems, USA) according to the manufacturer’s instructions, using the following primary antibodies: anti-PCNA (PC10, Dako, Denmark, 1:1200); anti-cyclin D1 (EP12, Dako, 1:200); anti-nm23 (4B2, Abzoom, USA, 1:200); and anti-Ki67 (MIB-1, Dako, 1:200). The IHC results were assessed by a pathologist blinded to the clinical outcome or histopathological diagnosis. PCNA, CCND1, and Ki67 immunoreactivities were restricted to the nucleus, while nm23 immunoreactivity was found in the cytoplasm. The Ki67 and PCNA indexes were obtained by counting, under a microscope, 1000 tumor cells in consecutive high-power fields in the most reactive areas, and determining (as a percentage) the number of these cells showing distinct nuclear staining. Scoring of CCND1 and nm23 expression was based on the intensity of the IHC staining and the percentage of positively stained cancerous cells, as described previously [15, 16]. Nuclear CCND1 immunostaining and cytoplasmic nm23 immunostaining was considered as positive. Scoring was as follows: 0, no staining; ±, focal, weak staining; 1+, weak staining in < 50 % of cells; 2+, weak staining in > 50 % of cells or strong staining in < 50 % of cells; 3+, strong staining in > 50 % of cells.

### Statistical analysis

A generalized linear model (GLM) in R [17] was applied to test for effects of the clinical, pathological, and IHC factors on SUVmax. In addition, for significant factors, a multivariate regression analysis was applied in PAST [18] to test the correlations between the significant factors and SUVmax. Receiver operating characteristics (ROC) analysis with calculation of the Youden index was used to determine the optimal SUVmax cutoff value for predicting the outcome of colorectal cancer. Kaplan–Meier survival curves were constructed to compare survival between patients with SUVmax values either side of this cutoff, with statistical comparisons made using the log-rank (Mantel-Cox) test. *P* < 0.05 was taken to indicate a statistically significant difference.

## Results

### Baseline patient characteristics

A total of 107 patients were included in the analysis: 77 in the survivor group and 30 in the deceased group. There were no significant differences between the two groups for age or gender (Table 1).

**Table 1:** Baseline patient data and univariate analysis of factors associated with survival at 60 months

Type	Group	Subgroup	Survivor (*N* = 77)	Deceased (*N* = 30)	*P* value
Demographic data	Age (years)		59.65 ± 12.75	58.27 ± 12.58	0.614
	Gender	Male	50 (64.9 %)	21 (70 %)	0.618*
		Female	27 (35.1 %)	9 (30 %)	
Pathologic factors	Colorectum	Colon cancer	45 (58.4 %)	15 (50 %)	0.389*
		Rectal cancer	31 (40.3 %)	15 (50 %)	
	Tumor size (cm)		5.0 (2.0, 10.0)	3.7 (1.0, 10.0)	0.017
	TNM stage	Tis	2 (2.6 %)	0	<0.001*
		I	15 (19.5 %)	0	
		IIA	21 (27.3 %)	1 (3.3 %)	
		IIB	10 (13.0 %)	2 (6.7 %)	
		III	24 (31.2 %)	14 (46.7 %)	
		IV	5 (6.5 %)	13 (43.3 %)	
	Histologic type	Adenocarcinoma	67 (87.0 %)	26 (86.7 %)	0.436*
		Mucinous adenocarcinoma	3 (3.9 %)	3 (10.0 %)	
		Signet ring cell carcinoma	1 (1.3 %)	0	
		Adenocarcinoma with component of mucinous adenocarcinoma	3 (3.9 %)	1 (3.3 %)	
		Adenocarcinoma with component of signet ring cell carcinoma	1 (1.3 %)	0	
		Other	2 (2.6 %)	0	
	Differentiation degree	Well differentiated	4 (5.2 %)	0	0.054*
		Well or moderately differentiated	4 (5.2 %)	0	
		Moderately differentiated	54 (70.1 %)	24 (80.0 %)	
		Moderately or poorly differentiated	8 (10.4 %)	3 (10.0 %)	
		Poorly differentiated	5 (6.5 %)	1 (3.3 %)	
		Undifferentiated	1 (1.3 %)	1 (3.3 %)	
	Nodal metastasis		0 (0,12)	2 (0,9)	0.01
	Ratio of nodal metastasis to retrieved nodes		0 % (0 %, 100 %)	8.93 % (0, 100)	0.03
Immunohistochemical factors	PCNA		60 % (0 %, 95 %)	52.5 % (10, 85)	0.129
	nm23	0 = "(–)"	9 (11.7 %)	5 (16.7 %)	0.65*
		1 = "(±)"	11 (14.3 %)	2 (6.7 %)	
		2 = "(+)"	51 (66.2 %)	22 (73.3 %)	
		3 = "(++)"	2 (2.6 %)	0	
		4 = "(+++)"	4 (5.2 %)	1 (3.3 %)	
	Cyclin D1	0 = "(–)"	14 (18.2 %)	3 (10.0 %)	0.03*
		1 = "(±)"	28 (36.4 %)	5 (16.7 %)	
		2 = "(+)"	31 (40.3 %0	15 (50.0 %)	
		3 = "(++)"	3 (3.9 %)	6 (20.0 %)	
		4 = "(+++)"	1 (1.3 %)	1 (3.3 %)	
	Ki67		50 (0,95)	45 (5,90)	0.908
Maximum standardized uptake value	SUVmax		9.8 (4.4, 24.7)	13.6 (3.2, 34.2)	<0.001

There is one missing number in “Colon or Rectal cancer” in the survivor group. And each group had a missing in “Differentiation degree”

*Means data was shown in number (percentage) and analyzed by Chi-square test. And the other data are all shown in median (minimum, maximum) and analyzed by ANOVA

### Univariate analysis of the factors associated with patient survival

In the univariate analysis, the pathologic factors significantly associated with survival were tumor size, TNM stage, nodal metastasis, and the ratio of metastasized nodes to retrieved nodes (all *P* < 0.05; Table 1). In contrast, lesion location, histologic type, and the degree of differentiation were not significantly associated with survival. Of the IHC factors assessed, only CCND1 was significantly associated with survival (*P* < 0.05); PCNA, nm23, and Ki67 showed no significant association (Table 1). SUVmax was also associated with patient survival (*P* < 0.05; Table 1).

### Multivariate analysis of the factors associated with patient survival

All factors showing a significant association with survival in the univariate analyses (*P* < 0.05) were entered into a multivariate analysis. The multivariate analysis revealed that only the TNM stage and SUVmax were independently associated with survival (*P* < 0.05), whereas tumor size, nodal metastasis, the ratio of metastasized nodes to retrieved nodes, and CCND1 were not (Table 2).

**Table 2:** Multivariate analysis of factors associated with survival at 60 months

	B	S.E.	Wald	df	Sig.	Exp(B)	95 % C.I. for Exp(B)	
							Lower	Upper
TNM stage	1.522	0.425	12.82	1	< 0.001	4.581	1.991	10.538
Tumor size	−0.006	0.180	0.001	1	0.975	0.994	0.699	1.414
SUVmax	0.261	0.091	8.268	1	0.004	1.298	1.087	1.551
Nodal metastasis	0.261	0.238	1.209	1	0.272	1.299	0.815	2.070
Ratio of nodal metastasis to retrieved nodes	−0.029	0.028	1.052	1	0.305	0.972	0.919	1.027
Cyclin D1	−0.293	0.423	0.480	1	0.488	0.746	0.325	1.710
Constant	−9.458	2.275	17.282	1	0.000	0.000		

A multivariate regression analysis was applied in PAST to test the correlations between the significant factors and SUVmax. And only TNM stage and SUVmax are independent risk factors for prognosis of colorectal cancer

### ROC analysis of the optimal SUVmax cutoff value for predicting survival in patients with colorectal cancer

**Fig. 1:**
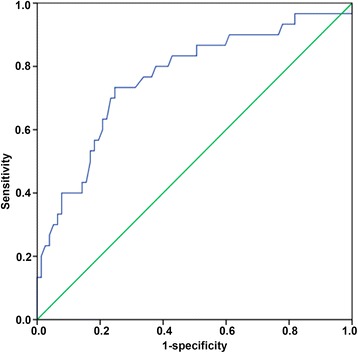
ROC curve analysis of the optimal SUVmax cutoff for predicting survival in patients with colorectal cancer

ROC analysis (Fig. 1) and calculation of the Youden index revealed that the optimal SUVmax cutoff value for predicting survival in patients with colorectal cancer was 11.85 (area under the curve, 0.763; *P* < 0.001). The calculated sensitivity and specificity values for this cutoff were 73.3 and 75.3 %, respectively.

**Fig. 2:**
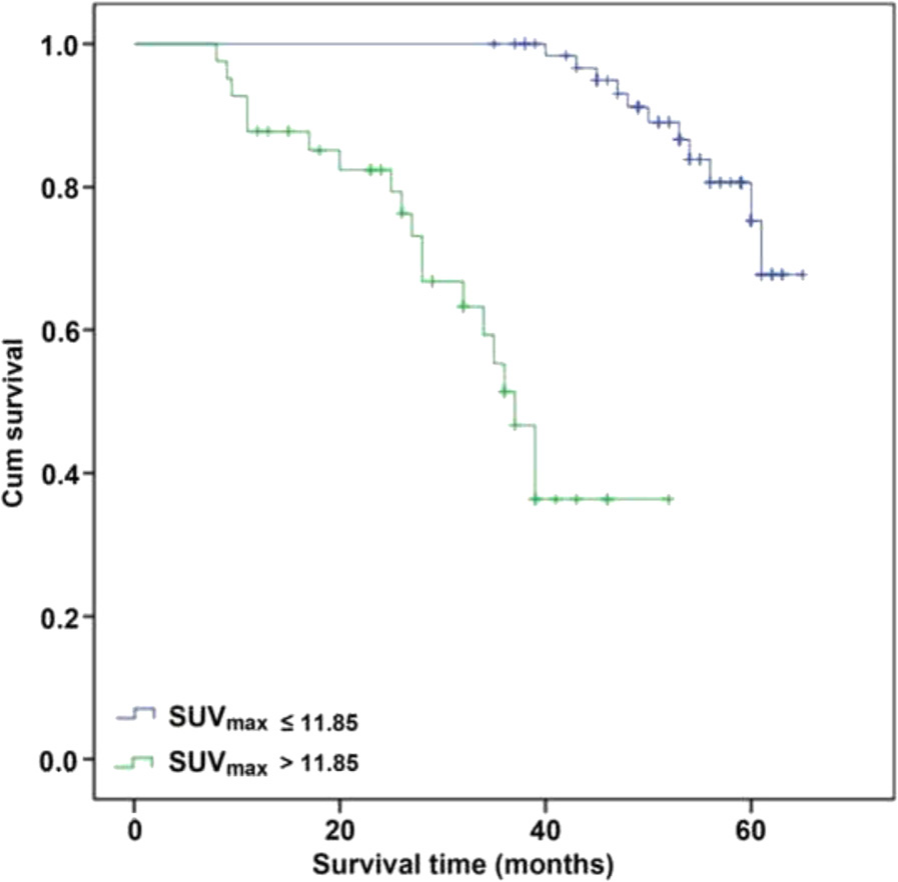
Comparison of survival curves for patients with SUVmax ≤ 11.85 and patients with SUVmax > 11.85

Figure 2 compares the Kaplan–Meier survival curves between patients with SUVmax ≤11.85 and those with SUVmax >11.85. Survival was significantly longer in patients with SUVmax ≤11.85 (*P* < 0.001). The median survival time was 37 months in patients with SUVmax >11.85, whereas median survival was not reached (i.e. exceeded 60 months) in patients with SUVmax ≤11.85.

### The correlation between IHC factors and SUVmax

**Fig. 3:**
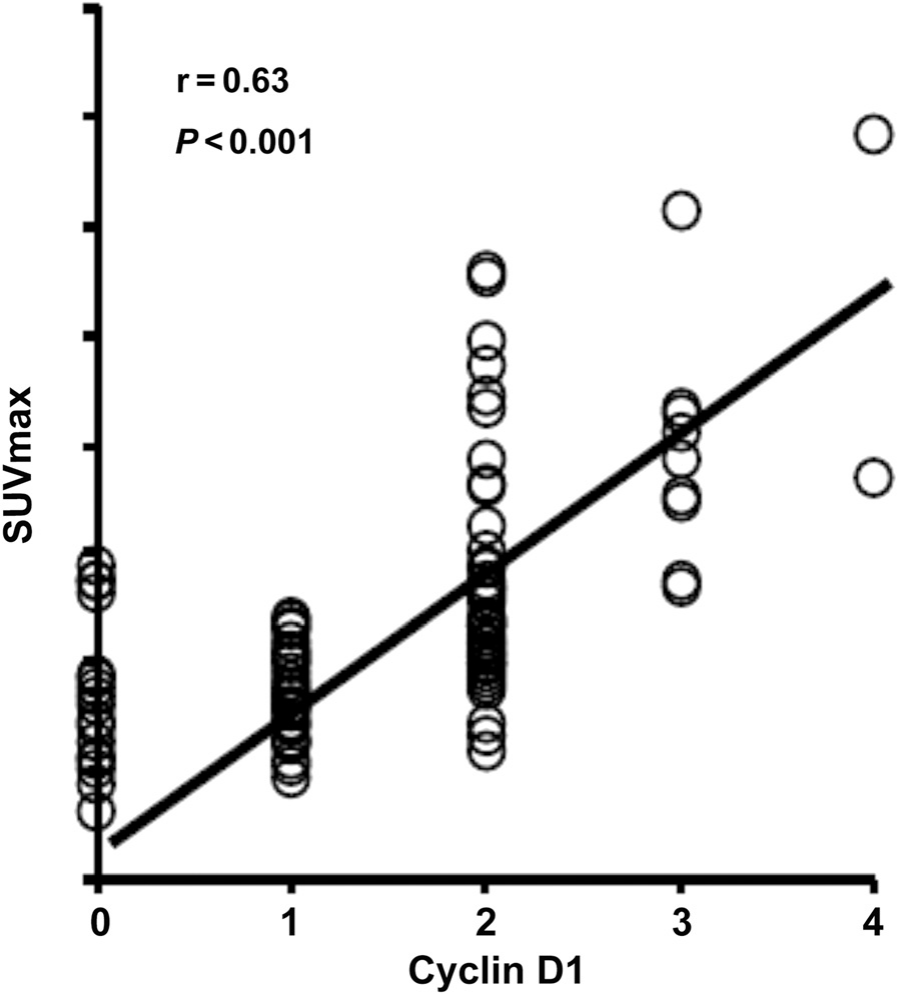
Correlation between cyclin D1 expression and SUVmax

Regression analysis indicated that SUVmax showed a significant positive correlation with the CCND1 immunostaining score (Fig. 3; r = 0.63; *P* < 0.001). However, SUVmax showed no significant correlations with immunostaining for PCNA, Ki67, or nm23 (data not shown).

### Case study

**Fig. 4:**
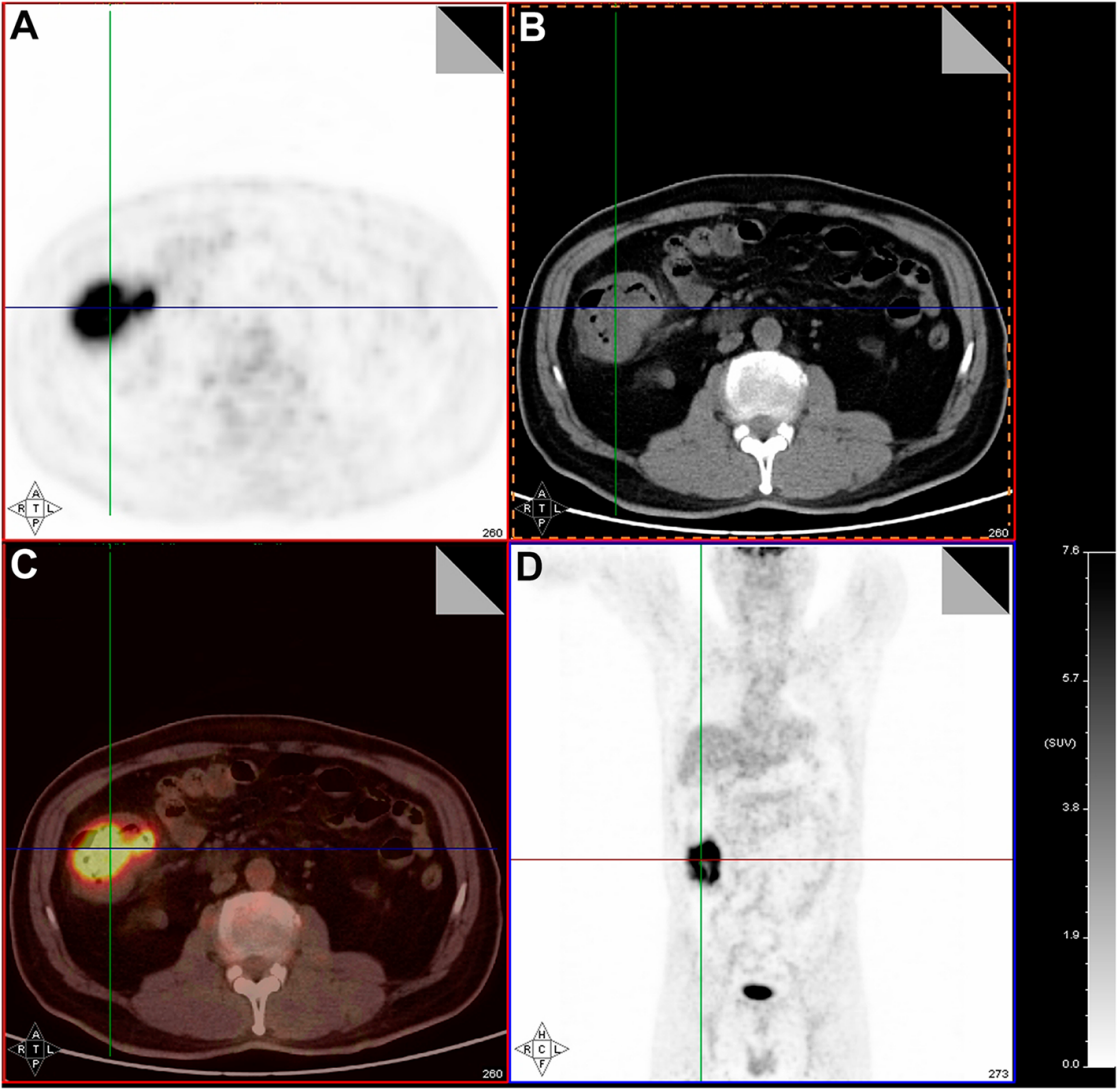
Case study of a patient with ascending colon cancer. **a** Transaxial PET image showing focal FDG uptake in the ascending colon, taken at the level indicated by the line. **b** Transaxial CT image of the same lesion. **c** Image obtained by merger of the PET and CT images. **d** Coronal PET image of the same lesion

A 58-year-old male with ascending colon cancer is described as a case study, to explore the correlations between clinicohistopathological factors and ^18^F-FDG uptake of the primary tumor. Representative PET and CT images are shown in Fig. 4. The transaxial PET image (Fig. 4a) indicated that focal FDG uptake in the ascending colon (i.e. SUVmax) was 12.8, while the transaxial CT image of the same lesion (Fig. 4b) showed that the tumor size was 6 × 8 cm. The merged PET/CT images (Fig. 4c) showed good correspondence. A coronal PET image of the same lesion is presented in Fig. 4d.

## Discussion

The main findings of this study are that higher TNM stage and higher SUVmax are significantly associated with shorter survival in patients with colorectal cancer, and that SUVmax is a marker of prognosis in these patients. Specifically, we determined that the optimal SUVmax cutoff value for predicting survival was 11.85, with values above 11.85 being associated with significantly shorter survival. Therefore, the measurement of SUVmax with ^18^F-FDG-PET/CT scanning provides a useful preoperative prognostic factor for patients with colorectal cancer. If the cutoff value is changed to 17, then the sensitivity would be of 75 % (18/24) for predicting death, which might suggest that a small proportion (22.4 %, 24/107) of patients would receive intensive treatments and that most of them (75 %) would still benefit from the treatment.

^18^F-FDG PET/CT imaging has been employed widely to identify and stage various types of cancer, including lung cancer [19], breast cancer [20], esophageal cancer [21], sarcoma [22], and melanoma [23], and has proven particularly useful in the detection, staging, and surveillance of colorectal cancer [24–27]. Accurate preoperative visualization of cancer deposits with this technique potentially enables surgeons to perform more complete tumor resections, improving clinical care and long-term survival. Semi-quantitative analysis with ^18^F-FDG PET/CT is gaining popularity for predicting clinical outcomes and determining the tumor response to treatment [28, 29], since changes in tumor metabolism may be observed prior to changes in tumor size. Thus, a significant change in tumor SUV potentially could be used as a measure of the metabolic response of the tumor to therapy. Our findings extend the potential clinical utility of SUVmax measurements, suggesting that in Chinese patients with colorectal cancer, preoperative values ≤11.85 can be used as a prognostic indicator of improved survival after surgery. This can provide additional guidance to clinicians treating patients with this cancer.

However, ^18^F-FDG PET/CT imaging does have some limitations including false-negative findings that can occur for several reasons (e.g., inflammation, small lesion size, and diabetes). Weston et al. found that the sensitivity, specificity, and accuracy of PET/CT at detecting colon cancer or adenomas >10 mm were 72, 90 and 88 %, respectively [28]. Sarikaya et al. [30] reported that 3 of 5 patients (60 %) with false-negative PET/CT findings had mucinous adenocarcinoma diagnosed histologically. Peng et al. have shown that colonoscopy is a necessity when incidental colorectal FDG uptake is found on ^18^F-FDG PET/CT imaging [31]. These authors also reported that the SUVmax value was higher in patients with cancer, although a high value did not necessarily indicate the presence of malignancy.

Regional lymph node status is an important prognostic factor that also plays a crucial role in the selection of postoperative therapy. Preoperative nodal staging using imaging requires an assessment of the number of pericolic and mesenteric nodes that contain metastatic disease. False-negative PET findings in regional metastatic lymph nodes are not uncommon, occurring in part due to the intense FDG uptake by the primary tumor that obscures immediately adjacent structures, and in part due to the low sensitivity of PET to microscopically involved lymph nodes. As a result, FDG PET has been found to have a high specificity (>90 %) for regional lymph node metastases from colorectal cancer, but only a low sensitivity (<30 %) [32, 33]. Yu et al. have reported recently that an SUVmax cutoff of 2.0 could identify malignant juxtaintestinal lymph nodes with a sensitivity of 91.43 % and a specificity of 87.83 % [34], highlighting the benefits of using SUVmax with ROC curve analysis for optimizing the diagnostic capabilities of ^18^F-FDG PET/CT.

It was notable that our multivariate analysis highlighted SUVmax and TNM stage as the two variables significantly associated with survival. Cancer stage is well established as a prognostic factor for colorectal cancer [35]. Although univariate analysis revealed that tumor size is correlated with survival, multivariate analysis found no significant influence of this factor in Chinese patients; this is in contrast to a recent study that reported tumor size to be an independent prognostic factor in Austrian patients with colon carcinoma [36]. Whether this reflects an ethnicity-related difference remains to be determined.

There is some debate as to the utility of Ki-67, PCNA, CCND1, and nm23 expressions as prognostic factors in colorectal cancer. Several studies have suggested that the expressions of these proteins correlate with outcome [11–13], whereas others have provided contradictory data [37–40]. The reasons underlying these discrepancies remain unknown. The multivariate analysis in the present study revealed that the expressions of Ki-67, PCNA, CCND1, and nm23 did not correlate significantly with survival, suggesting that in Chinese patients, preoperative IHC assessments of these markers may not be particularly useful indicators of prognosis after surgery. Interestingly, we did find a significant correlation between SUVmax and CCND1 expression; the underlying reasons for this correlation merit further study.

The present study is not without limitations. First, this was a hospital-based study; hence, our patients may not be sufficiently representative of the general population in China. Second, a relatively small number of patients were included, raising the possibility of inherent selection bias. Third, our study did not include a healthy control group for comparison. Fourth, our study was retrospective in nature. Finally, these patients received a wide variety of adjuvant treatments that could not be taken into account in the analyses. Therefore, larger case-control and prospective randomized studies are needed to validate and extend our findings.

## Conclusions

In conclusion, TNM stage and SUVmax are independent predictors of survival in patients with colorectal cancer, and preoperative SUVmax values ≤11.85 are associated with better survival. FDG-PET/CT could be used as a method of patient stratification before surgery, helping in the selection of appropriate therapeutic strategies.

## Abbreviations

^18^F-FDG: ^18^F-fluorodeoxyglucose
CCND1: cyclin D1
CT: computed tomography
GLM: generalized linear model
IHC: immunohistochemical
NCCN: National Comprehensive Cancer Network
PCNA: proliferating cell nuclear antigen
PET: positron emission tomography
ROC: receiver operating characteristics
SUV: standardized uptake value
SUVmax: maximal standardized uptake value

## References

[1] Jemal A, Siegel R, Ward E, Hao Y, Xu J, Murray T (2008). Cancer statistics, 2008. CA Cancer J Clin..

[2] Shanghai Municipal Center for Disease Control and Prevention (2007). Shanghai Cancer Report.

[3] Van Cutsem E, Nordlinger B, Adam R, Kohne CH, Pozzo C, Poston G (2006). Towards a pan-European consensus on the treatment of patients with colorectal liver metastases. Eur J Cancer..

[4] Yoo PS, Lopez-Soler RI, Longo WE, Cha CH (2006). Liver resection for metastatic colorectal cancer in the age of neoadjuvant chemotherapy and bevacizumab. Clin Colorectal Cancer..

[5] Pihl E, Hughes ES, McDermott FT, Johnson WR, Katrivessis H (1987). Lung recurrence after curative surgery for colorectal cancer. Dis Colon Rectum..

[6] Foster JH (1984). Treatment of metastatic disease of the liver: a skeptic's view. Semin Liver Dis..

[7] O'Connor OJ, McDermott S, Slattery J, Sahani D, Blake MA (2011). The Use of PET-CT in the Assessment of Patients with Colorectal Carcinoma. Int J Surg Oncol..

[8] Farquharson AL, Chopra A, Ford A, Matthews S, Amin SN, De Noronha R (2012). Incidental focal colonic lesions found on (18)Fluorodeoxyglucose positron emission tomography/computed tomography scan: further support for a national guideline on definitive management. Colorectal Dis..

[9] Jadvar H, Alavi A, Gambhir SS (2009). 18 F-FDG uptake in lung, breast, and colon cancers: molecular biology correlates and disease characterization. J Nucl Med..

[10] Schneider NI, Langner C (2014). Prognostic stratification of colorectal cancer patients: current perspectives. Cancer Manag Res..

[11] Guzinska-Ustymowicz K, Pryczynicz A, Kemona A, Czyzewska J (2009). Correlation between proliferation markers: PCNA, Ki-67, MCM-2 and antiapoptotic protein Bcl-2 in colorectal cancer. Anticancer Res..

[12] Wu HW, Gao LD, Wei GH (2013). hMSH2 and nm23 expression in sporadic colorectal cancer and its clinical significance. Asian Pac J Cancer Prev..

[13] Bahnassy AA, Zekri AR, El-Houssini S, El-Shehaby AM, Mahmoud MR, Abdallah S (2004). Cyclin A and cyclin D1 as significant prognostic markers in colorectal cancer patients. BMC Gastroenterol..

[14] Kikuchi M, Mikami T, Sato T, Tokuyama W, Araki K, Watanabe M (2009). High Ki67, Bax, and thymidylate synthase expression well correlates with response to chemoradiation therapy in locally advanced rectal cancers: proposal of a logistic model for prediction. Br J Cancer..

[15] Tsai HL, Yeh YS, Chang YT, Yang IP, Lin CH, Kuo CH (2013). Co-existence of cyclin D1 and vascular endothelial growth factor protein expression is a poor prognostic factor for UICC stage I-III colorectal cancer patients after curative resection. J Surg Oncol..

[16] Delektorskaya VV, Perevoshchikov AG, Kushlinskii NE (2003). Immunohistological study of NM 23 and C-erbB-2 expression in primary tumor and metastases of colorectal adenocarcinoma. Bull Exp Biol Med..

[17] Statistical PR (2009). R: A language and environment for statistical computing.

[18] Hammer Ø, Harper D, Ryan P. Past: Paleontological Statistics Software Package for education and data analysis. Paleontología Electrónica 4: 1-9. URL: http://palaeo-electronica.org/2001_1/past/issue1_01.htm. 2001.

[19] Pieterman RM, van Putten JW, Meuzelaar JJ, Mooyaart EL, Vaalburg W, Koeter GH (2000). Preoperative staging of non-small-cell lung cancer with positron-emission tomography. N Engl J Med..

[20] Evangelista L, Cervino AR, Michieletto S, Saibene T, Orvieto E, Bozza F et al. Staging of locally advanced breast cancer and the prediction of response to neoadjuvant chemotherapy: complementary role of scintimammography and 18F-FDG PET/CT. Q J Nucl Med Mol Imaging. 2014. 10.23736/S1824-4785.16.02741-2 25501326

[21] Hsu WH, Hsu PK, Wang SJ, Lin KH, Huang CS, Hsieh CC (2009). Positron emission tomography-computed tomography in predicting locoregional invasion in esophageal squamous cell carcinoma. Ann Thorac Surg..

[22] Johnson GR, Zhuang H, Khan J, Chiang SB, Alavi A (2003). Roles of positron emission tomography with fluorine-18-deoxyglucose in the detection of local recurrent and distant metastatic sarcoma. Clin Nucl Med..

[23] Bastiaannet E, Oyen WJ, Meijer S, Hoekstra OS, Wobbes T, Jager PL (2006). Impact of [18 F]fluorodeoxyglucose positron emission tomography on surgical management of melanoma patients. Br J Surg..

[24] Akhurst T, Larson SM (1999). Positron emission tomography imaging of colorectal cancer. Semin Oncol..

[25] Sanli Y, Kuyumcu S, Ozkan ZG, Kilic L, Balik E, Turkmen C (2012). The utility of FDG-PET/CT as an effective tool for detecting recurrent colorectal cancer regardless of serum CEA levels. Ann Nucl Med..

[26] Han A, Xue J, Zhu D, Zheng J, Yue J, Yu J (2011). Clinical value of (18)F-FDG PET/CT in postoperative monitoring for patients with colorectal carcinoma. Cancer Epidemiol..

[27] Chen LB, Tong JL, Song HZ, Zhu H, Wang YC (2007). (18)F-DG PET/CT in detection of recurrence and metastasis of colorectal cancer. World J Gastroenterol.

[28] Weston BR, Iyer RB, Qiao W, Lee JH, Bresalier RS, Ross WA (2010). Ability of integrated positron emission and computed tomography to detect significant colonic pathology: the experience of a tertiary cancer center. Cancer..

[29] Andersen KF, Skougaard K, Nielsen AL, Hendel HW (2012). Impact of third-line treatment with irinotecan plus cetuximab on non-tumor standardized uptake values in patients with metastatic colorectal cancer. Oncol Lett..

[30] Sarikaya I, Bloomston M, Povoski SP, Zhang J, Hall NC, Knopp MV (2007). FDG-PET scan in patients with clinically and/or radiologically suspicious colorectal cancer recurrence but normal CEA. World J Surg Oncol..

[31] Peng J, He Y, Xu J, Sheng J, Cai S, Zhang Z (2011). Detection of incidental colorectal tumours with 18 F-labelled 2-fluoro-2-deoxyglucose positron emission tomography/computed tomography scans: results of a prospective study. Colorectal Dis..

[32] Abdel-Nabi H, Doerr RJ, Lamonica DM, Cronin VR, Galantowicz PJ, Carbone GM (1998). Staging of primary colorectal carcinomas with fluorine-18 fluorodeoxyglucose whole-body PET: correlation with histopathologic and CT findings. Radiology..

[33] Mukai M, Sadahiro S, Yasuda S, Ishida H, Tokunaga N, Tajima T (2000). Preoperative evaluation by whole-body 18 F-fluorodeoxyglucose positron emission tomography in patients with primary colorectal cancer. Oncol Rep..

[34] Yu L, Tian M, Gao X, Wang D, Qin Y, Geng J (2012). The method and efficacy of 18 F-fluorodeoxyglucose positron emission tomography/computed tomography for diagnosing the lymphatic metastasis of colorectal carcinoma. Acad Radiol..

[35] O'Connell JB, Maggard MA, Ko CY (2004). Colon cancer survival rates with the new American Joint Committee on Cancer sixth edition staging. J Natl Cancer Inst..

[36] Kornprat P, Pollheimer MJ, Lindtner RA, Schlemmer A, Rehak P, Langner C (2011). Value of tumor size as a prognostic variable in colorectal cancer: a critical reappraisal. Am J Clin Oncol..

[37] Fodor IK, Hutchins GG, Espiritu C, Quirke P, Jubb AM (2012). Prognostic and predictive significance of proliferation in 867 colorectal cancers. J Clin Pathol..

[38] McKay JA, Douglas JJ, Ross VG, Curran S, Loane JF, Ahmed FY (2002). Analysis of key cell-cycle checkpoint proteins in colorectal tumours. J Pathol..

[39] Crowe PJ, Yang JL, Berney CR, Erskine C, Ham JM, Fisher R (2001). Genetic markers of survival and liver recurrence after resection of liver metastases from colorectal cancer. World J Surg..

[40] Ioachim E (2008). Expression patterns of cyclins D1, E and cyclin-dependent kinase inhibitors p21waf1/cip1, p27kip1 in colorectal carcinoma: correlation with other cell cycle regulators (pRb, p53 and Ki-67 and PCNA) and clinicopathological features. Int J Clin Pract..

